# Teaching residents in emergency surgery for acute bowel obstruction—is supervised surgery safe and feasible? A retrospective single-center analysis from a MIS-specialized hospital

**DOI:** 10.1007/s00464-024-11410-9

**Published:** 2024-12-02

**Authors:** Hannes Hoi, Barbara Ebner, Martin Grünbart, Michael de Cillia, Robert Uzel, Lisa Schlosser, Helmut Weiss, Christof Mittermair

**Affiliations:** 1 Department of General and Visceral Surgery, St. John of God Hospital, Teaching Hospital of the Paracelsus Medical University Salzburg, Kajetanerplatz 1, 5010 Salzburg, Austria; 2 Department of Radiology, St. John of God Hospital, Teaching Hospital of the Paracelsus Medical University Salzburg, Kajetanerplatz 1, 5010 Salzburg, Austria; 3 Department of Internal Medicine, St. John of God Hospital, Teaching Hospital of the Paracelsus Medical University Salzburg, Kajetanerplatz 1, 5010 Salzburg, Austria; 4 Department of Mathematics, University of Innsbruck, Technikerstrasse 13, 6020 Innsbruck, Austria

**Keywords:** Acute bowel obstruction, Teaching and supervision, Minimally invasive surgery, Open surgery

## Abstract

**Objective:**

Emergency surgery for acute bowel obstruction (ABO) is a common and occasionally technically demanding procedure, requiring both surgical skill and strategic planning. The risk entailed in teaching residents during ABO surgery has not been defined or investigated in detail to date. It is the aim of this study to reveal whether surgery for ABO, performed by resident surgeons under supervision, is safe and feasible.

**Design:**

A retrospective analysis was conducted of all emergency surgeries for ABO performed between 2009 and 2023 at a community-based hospital. Patients’ general characteristics, procedural data and outcome parameters were compared. Differences between teaching procedures and non-teaching procedures were analysed.

**Setting:**

The study was conducted at the Department of General and Visceral surgery at a community-based hospital (St. John of God Hospital Salzburg, Austria).

**Participants:**

All emergency surgeries for ABO (*n* = 300 patients) that were performed during the study period were included.

**Results:**

Emergency surgery for ABO was performed in 300 patients during the study period, 15.3% of which operations were performed by residents under supervision and 84.7% by senior surgeons. No differences between these two groups were found in terms of patient characteristics, except for a past medical history of previous gynecologic or urologic surgery that was more frequent in the senior surgeon group (*p* = 0.02). Neither procedural data nor conversion rates from a minimally invasive (MIS) to an open (OS) approach, nor postoperative complication rates were found to be significantly different between these groups.

**Conclusion:**

Emergency surgery for ABO, performed by residents under supervision, is safe and feasible, showing no significant differences in terms of complication rates, morbidity or mortality as compared to procedures performed by senior surgeons.

**Graphical abstract:**

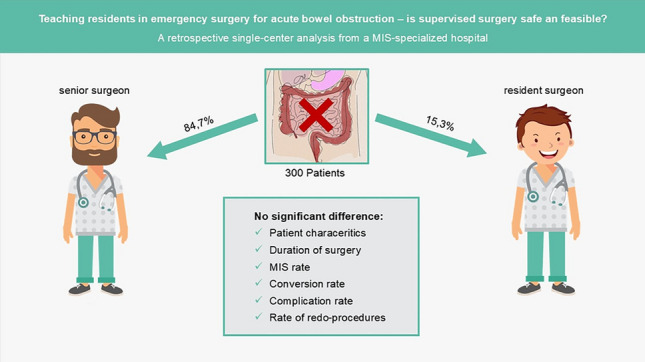

ABO is known to be one of the most common causes for abdominal emergency surgery in hospitals all over the world [1]. Therefore, adequate teaching and supervision in the surgical management of this pathology should be of utmost importance for surgical residents and young consultants.

Dimensions of teaching surgical procedures not only include repeated on-table assistance, but also simulation and perioperative briefing and debriefing that are known to flatten the individual learning curve [2]. However, teaching is not limited to passing on technical skills, but also includes strategy development and perioperative management. In a second step of surgical education, it is necessary that procedures be self-performed by residents/trainees under supervision in order to pave the way to their becoming independent surgeons. [3]

Especially the surgical management of ABO can be technically demanding, depending on the cause of the obstruction, ranging from a single band adhesion to a frozen abdomen or obstructing tumour. This requires the presence of an experienced surgeon.

In times of reduced core working hours the number of surgical procedures performed by residents is declining [4]. Moreover, emergency procedures are mostly performed outside core working hours and therefore do not underlie a certain planning reliability, making it potentially difficult for surgeons to achieve a sufficient case load before being responsible for surgery performed independently in ABO patients. The aforementioned factors require a preponement of these procedures to an earlier phase of surgical training in order to acquire adequate professional experience.

As a result of the lack of standardization and guidelines for the individual surgical management of ABO, little is known about the safety and feasibility of training andteaching in this field. Although there are standardized protocols and widely available learning courses for young surgeons to optimally improve their skills in the management of various procedures such as appendectomy or cholecystectomy, this kind of support is not available for ABO surgery [5, 6]. Only the Bologna guidelines, published in 2018 by Ten Broek et al., provide a panel of recommendations for diagnosing and dealing with ABO patients, but offer only vague suggestions when it comes to surgical-technical and strategic considerations [7]. Regarding the complexity of different factors influencing surgery for ABO, comprising patient comorbidities, past medical history, advanced surgical skills and the need for individually adjusted decision-making, it is generally considered very difficult to routinely incorporate this type of surgery in a standardized teaching program for surgical residents.

It was the aim of this study to examine whether ABO surgery performed by residents under supervision is safe and feasible.

## Material and methods

### Study design and setting

This retrospective analysis was conducted at the Department of Surgery of Saint John of God Hospital in Salzburg (Austria) and included all consecutive emergency surgeries for ABO that were performed between January 1, 2009 and December 12, 2022. All surgical procedures were performed by either a certified surgical expert (senior surgeon) or a surgical resident under the supervision of a specialist. The hospital’s surgical team consists of seven surgical specialists and an alternating number of two to five residents in their first to sixth year of training. All specialists have performed a minimum of 100 advanced MIS (colorectal and upper GI) and more than 100 complex OS procedures. The decision for surgery was always made by a surgical specialist (senior surgeon), who was allowed to liberally decide the surgical approach of choice (MIS or OS), although the hospital’s surgical department has a special focus on advanced MIS.

### Inclusion and exclusion criteria

All emergency surgeries for ABO that were consecutively performed during the study period were retrospectively analysed. Patients aged ≤ 15 years and pregnant patients were excluded from the study, as the hospital does not cover the requirements for a paediatric or obstetric medical care unit.

### Data acquisition/measurements

Data were collected in the hospital-intern software system “Patidok 2.0” and exported to a Microsoft Excel database (Microsoft Excel, Microsoft Corporation, Redmond, Washington (WA), USA). Data analysis comprised patient characteristics (age, gender, BMI, ASA classification, bowel dilatation in cm in radiologic imaging, inflammatory parameters (CRP), type and number of previous abdominal surgeries), procedural data (time when surgery was begun, duration of surgery, operating and assisting surgeon, reason for ABO/intraoperative findings, MIS or OS approach) and outcome parameters (length of stay (LOS), need and reason for conversion, complication rates, rates of redo-surgeries).

The dataset was split into two groups: Group 1 comprised all surgeries performed by a resident under supervision of a specialist and Group 2 comprised all surgeries performed by a specialist (with either an assisting specialist, resident or intern).

A subgroup analysis was performed, comparing patient characteristics and outcome parameters between procedures performed by residents under supervision (Group 1) and those performed by specialists with an assisting resident (Subgroup 1).

### Outcome parameters

Complication and conversion rates were defined as primary outcome parameters. Patient characteristics and procedural data served as secondary outcome parameters.

### Analysis

Statistical analysis was performed by a mathematician not involved in patient assessment (L.S.) using R, version 4.0.5. All statistical calculations were two-sided and a significance level of 5% was applied. Group differences were assessed with the Wilcoxon rank sum test for continuous variables and Fisher’s exact test for binary variables. Continuous data are presented as median (25th to 75th percentile) and categorical variables as frequencies (%). Effect size and precision are shown with estimated median differences between groups for continuous data and odds ratios (OR) for binary variables, with 95% confidence intervals (CIs).

## Theory/calculation

## Results

A total of 300 patients underwent emergency surgery for ABO between January 1, 2009 and December 12, 2022. Of these, 46 (15.3%) procedures were performed by a resident under the supervision of a surgical specialist (Group 1) and 254 (84.7%) procedures were performed by a surgical specialist (Group 2; with either an assisting senior surgeon, resident or intern). Patient characteristics and past medical history did not significantly differ between the groups. Only patients who had a past medical history of previous gynecologic or urologic surgery were more likely to undergo surgery performed by a senior surgeon (*p* = 0.02). Results are shown in Table 1.

**Table 1:** Patient characteristics in ABO surgeries in Group 1 (resident under supervision) and Group 2 (senior surgeon)

	Total (*n* = 300)	Group 1 (*n* = 46)	Group 2 (*n* = 254)	Estimate with 95% CI^a^	*p* value^b^
Age	75.46 (61.02–81.64)	76.34 (57.4–81.23)	75.18 (61.97–81.64)	−0.43 (−6.62 to 5.11)	0.86
Gender					
Male	116/300 (38.7%)	14/46 (30.4%)	102/254 (40.2%)	0.65 (0.31 to 1.33)	0.25
Female	184/300 (61.3%)	32/46 (69.6%)	152/254 (59.8%)	0.65 (0.31 to 1.33)	0.25
BMI	23.4 (20.5–26.48)	23.4 (20.2–26.4)	23.4 (20.5–26.5)	−0.3 (−1.7 to 1)	0.63
ASA classification	2 (2–3)	2 (2–3)	2 (2–3)	0 (0 to 0)	0.61
Bowel width (maximum diameter in cm)	3.5 (3–4)	3.5 (3–4)	3.5 (3–4)	0 (−0.3 to 0.2)	0.69
CRP elevation yes/no					
Yes	171/300 (57%)	28/46 (60.9%)	143/254 (56.3%)	1.21 (0.61 to 2.44)	0.63
No	129/300 (43%)	18/46 (39.1%)	111/254 (43.7%)	1.21 (0.61 to 2.44)	0.63
Number of previous abdominal surgeries	1 (1–2)	1.5 (1–2.75)	1 (1–2)	0 (0 to 0)	0.96
Type of previous surgeries (MIS/OS)					
MIS	93/300 (31%)	14/46 (30.4%)	79/254 (31.1%)	0.97 (0.45 to 1.99)	1
OS	198/300 (66%)	32/46 (69.6%)	166/254 (65.4%)	1.21 (0.59 to 2.59)	0.62
Target organ/area of previous surgeries					
Appendix	84/300 (28%)	15/46 (32.6%)	69/254 (27.2%)	1.3 (0.61 to 2.65)	0.48
Colon	108/300 (36%)	11/46 (23.9%)	97/254 (38.2%)	0.51 (0.22 to 1.09)	0.07
Upper GI	88/300 (29.3%)	15/46 (32.6%)	73/254 (28.7%)	1.2 (0.57 to 2.45)	0.60
Urologic/Gynecologic	80/300 (26.7%)	19/46 (41.3%)	61/254 (24%)	2.22 (1.09 to 4.48)	0.02

^a^Binary data are presented as no./total no. (%), continuous data as medians (25th to 75th percentile)

^b^Odds ratios for binary variables and estimated median difference for continuous variables

^c^Assessed with Fisher’s exact test for categorical variables and with the Wilcoxon rank sum test for continuous variables

Concerning procedural data, the duration of surgery and the underlying reason for ABO did not differ between the two groups. In particular, with regard to the surgical approach, patients in Group 2 experienced a primary MIS rate of 47.8% compared to 53.1% in Group 1 patients (*p* = 0.53). All procedural data are shown in Table 2.

**Table 2:** Procedural data in ABO surgeries in Group 1 (resident under supervision) and Group 2 (senior surgeon)

	Total (*n* = 300)	Group 1 (*n* = 46)	Group 2 (*n* = 254)	Estimate with 95% CI^a^	*p* value^b^
Beginning of surgery (timepoint)	15 (12–18.75)	15.5 (13–18.75)	15 (11–18.25)	1 (− 1 to 2)	0.47
Duration of surgery (min)	68.5 (48–110)	71.5 (37.75–121.75)	67 (48–109.25)	2 (− 11 to 17)	0.82
Reason for ABO					
Adhesions	146/300 (48.7%)	26/46 (56.5%)	120/254 (47.2%)	1.45 (0.74 to 2.89)	0.27
Tumor (benign and malignant bowel and non-bowel neoplasms, metastatic disease)	23/300 (7.7%)	3/46 (6.5%)	20/254 (7.9%)	0.82 (0.15 to 2.94)	1
Other	67/300 (22.3%)	14/46 (30.4%)	53/254 (20.9%)	1.66 (0.76 to 3.47)	0.18

^a^Binary data are presented as no./total no. (%), continuous data as medians (25th to 75th percentile)

^b^Odds ratios for binary variables and estimated median difference for continuous variables

^c^Assessed with Fisher’s exact test for categorical variables and with the Wilcoxon rank sum test for continuous variables

Overall conversion rates yielded 8.7% and 18.9% in Group 1 and Group 2, respectively (*p* = 0.14). Reasons for conversion revealed no significant differences between the two groups. Neither postoperative complication rates nor the rate of redo-procedures differed significantly between the two groups (*p* = 0.73 and *p* = 0.55, respectively). 13 patients of the study cohort are duplicates and were readmitted (and treated surgically) to the hospital for recurrent bowel obstruction during the study period. Of those, only one patient was readmitted during the first 30 days after discharge to undergo surgery for recurrent ABO. Results of conversion and complication rates are shown in Table 3.

**Table 3:** Outcome parameters in ABO surgeries in Group 1 (resident under supervision) and Group 2 (senior surgeon)

	Total (*n*=300)		Group 1 (*n*=46)	Group 2 (*n*=254)	Estimate with 95% CI^a^	*p* value^b^
LOS	13 (8–23)		11.5 (8–23)	13 (8–22)	− 1 (− 3 to 2)	0.67
Conversions among started MIS	52/157 (33.1%)		4/22 (18.2%)	48/135 (35.6%)	0.4 (0.09 to 1.33)	0.14
Reason for conversion						
Exposure (among started MIS)	40/157 (25.5%)		4/22 (18.2%)	36/135 (26.7%)	0.61 (0.14 to 2.04)	0.60
Exposure (among conversions)	40/52 (76.9%)		4/4 (100%)	36/48 (75%)	Inf (0.19 to Inf)	0.56
Complication (among started MIS)	12/157 (7.6%)		12/135 (8.9%)	12/135 (8.9%)	0 (0 to 2.19)	0.22
Complication (among conversions)	12/52 (23.1%)		0/4 (0%)	12/48 (25%)	Inf (0.19 to Inf)	0.56
Complication (≥ Dindo 3b) yes / no						
Yes	97/300 (32.3%)		16/46 (34.8%)	81/254 (31.9%)	1.14 (0.55 to 2.3)	0.73
No	203/300 (67.7%)		30/46 (65.2%)	173/254 (68.1%)	1.14 (0.55 to 2.3)	0.73
Redo surgery						
Yes	61/300 (20.3%)		11/46 (23.9%)	50/254 (19.7%)	1.28 (0.55 to 2.81)	0.55
No	239/300 (79.7%)		35/46 (76.1%)	204/254 (80.3%)	1.28 (0.55 to 2.81)	0.55

^a^Binary data are presented as no./total no. (%), continuous data as medians (25th to 75th percentile)

^b^Odds ratios for binary variables and estimated median difference for continuous variables

^c^Assessed with Fisher’s exact test for categorical variables and with the Wilcoxon rank sum test for continuous variables

In 157 MIS procedures, pneumoperitoneum was established using the first port at the umbilicus, the left or right abdomen, the epigastric midline and the point of Mc Burney in 141, 12, two, one and one patients, respectively.

A subgroup analysis that should reveal differences between procedures performed by residents under supervision (Group 1) and procedures performed by senior surgeons with an assisting resident surgeon (Subgroup 1, *n* = 141 patients; with the intention that those procedures could have been taught to residents) revealed no significant differences in any of the investigated patient characteristics, procedural or outcome parameters. However, the rate of surgeries performed by residents was 24.6% in this patient cohort (46 out of 187 cases).

## Discussion

The results of this retrospective study show that even complex surgical procedures for the treatment of ABO are safe and feasible when performed by residents under supervision. Compared to emergency surgeries for ABO performed by specialists, supervised surgery revealed no differences in terms of patient characteristics, procedural or outcome parameters when performed by residents.

So far, only few scientific publications address the role of teaching in ABO surgery. The safety and feasibility of surgical teaching has been demonstrated for appendectomy, cholecystectomy, colorectal resections and for bariatric procedures. [14] [8–14] However, especially emergency procedures are underrepresented in these data, not least because of their unpredictable time of occurrence and high procedural variability. To name one of a few exceptional examples, a 2022 WSES (World Society of Emergency Surgery) position paper on minimally invasive emergency surgery revealed that proficiency was achieved after an average of 30 procedures (range: 20–107) [15]. However, the procedures investigated by these authors addressed mostly appendectomies. Regarding this and the results of the analysis presented herein, these findings emphasize the need for structured training of emergency procedures and its integration into the surgical residential curriculum.

The decision when to apply surgery or administer conservative treatment in patients with ABO is known to be difficult and both, patient- and surgeon-dependent [16]. In all study patients a senior surgeon indicated time of surgery based on patients symptoms, past medical history, radiological findings and laboratory results. Conservatively treated patients with no need for surgery were not included in this study.

It is of note that in our cohort only 15.3% of surgeries for ABO were teaching cases. The reasons for this might be the following: Firstly, surgeries for ABO were often performed outside core working hours, when only the surgical team on duty was available to perform the procedure. This team comprised two people, a senior surgeon together with a resident or an intern. With a resident on duty, the percentage of assisted procedures increased to 24.6% (46 out of 187 cases). Secondly, there is no clear department policy concerning when to teach or not to teach a surgical procedure. Important influencing factors that should be taken into consideration are the number of training years completed by the resident and the professional experience of the responsible specialist. Thirdly, insurance policies dictate that patients with private insurance have to be treated by certified surgeons.

Interestingly, no differences between the study populations, other than a past medical history of previous gynecologic or urologic surgery, were identified. This finding somehow contradicts the recommendation that surgical beginners should start with the best and easiest conditions (e.g. young, healthy patients with a low BMI). Nevertheless, this finding underlines the general willingness of the responsible surgeon to provide teaching and education for residents as long as it is feasible and safe.

Regarding the surgical approach, the MIS rate of 53.1% in the expert group did not significantly differ from the 47.8% in the resident group (*p* = 0.53), reflecting the growing awareness for and implementation of structured training of surgical skills in early residency. A comparable conclusion was made by Cullinan et al., who also revealed a steadily growing percentage of MIS procedures performed during residency [17]. This was confirmed by Malangoni et al., who also recognized an increase in MIS and endoscopic techniques at the expense of a decrease in open procedures in teaching operations. [18]

Concerning procedural time, the results of this analysis did not reveal any significant prolongation when residents served as primary surgeons. This stands partially in contrast to data from Lee et al. [9], who reported a significant prolongation in operating time and hospital stay for laparoscopic appendectomy when performed by residents. However, the same authors revealed no differences in terms of complications. A similar finding was published by Benissan-Messan et al., who investigated operating times for cholecystectomies, colectomies, and inguinal hernias [19]. These authors revealed an impact of resident training level, resident gender, and case complexity on procedural time and showed that operating time decreased with increased resident training level.

With respect to conversion from MIS to OS, a large nationwide retrospective analysis of nearly four million hospital admissions for ABO, containing data from 2001 to 2011, was published by Jafari et al. [20] This study revealed a MIS rate of only 26.5% and a low conversion rate of 22.5% [20]. However, more recent data from 2020 and 2022 revealed MIS rates of 52.6% and 32.7% with conversion rates of 47.4% and 36.7%, respectively. [21, 22] These findings are only partly in line with our own results that showed a MIS rate of 53.1% with a conversion rate of 18.9% in the expert group and a MIS rate of 47.8% with a conversion rate of 8.7% in the resident group (Group 1). These comparably low conversion rates in our own patient cohort are explained by the fact that the hospital is considered a centre for MIS and that a structured educational program for laparoscopic training already starts in early residency.

With respect to patient safety, complication rates (≥ Dindo 3b) were similar between the resident and the specialist group (34.8% and 31.9%). These substantially high rates, on the one hand, indicate that patients and procedures were comparable in both groups and, on the other hand, illustrate the critical state of health in the affected population. Moreover, similar results were found in an analysis of 269 surgeries for adhesive small bowel obstruction published by Byrne et al. [23] in 2015, where the authors revealed complication rates of 27.7% in the MIS group and 43.6% in the OS group.

Concerning redo surgery in the study cohort, a rate of 20.3% of redo procedures seems to be rather high when compared with results from literature: Figuera-Giralt et al. revealed a redo rate of only 9.2% in 431 patients with ABO [24]. Moreover, an analysis from 2023, including 366 patients, performed by Girón et al., showed a similar low redo rate of 9.5% [25]. However, authors focused on the rate of recurrent ABO rather than early postoperative redo due to bowel injury, anastomotic leakage or bleeding complications.

In summary, this study supports the intention to clear the way for future procedural standardization and guideline development when establishing a teaching strategy for ABO surgeries.

### Limitations

Since the study was conducted at a MIS-focused hospital, data may not reflect the average distribution among the percentages of MIS and OS techniques as compared to hospitals with no special focus on MIS.

The retrospective character of this study could be seen as a limitation. However, RCTs addressing teaching and supervision in this special surgical field would be difficult to perform and hardly feasible due to the emergency character of the pathology and due to the exceptional and very individual situations. Nevertheless, the department is already planning a prospective study design on this topic.

Since the character of this analysis is retrospective, we were not able to rule out selection bias that occurred when individual surgeries were either selected or refused as teaching procedures. Moreover, for legal reasons, patients with private insurance were treated by specialists only.

## Conclusions

Emergency surgery for ABO performed by residents under supervision is feasible and safe, resulting in outcomes similar to those for procedures performed by specialists. Structured training, mentorship and supervision as well as guidelines for the management of ABO are needed to facilitate the complex treatment of ABO for surgical residents and future surgeons.

## Data Availability

The datasets generated and analyzed in the current study are not publicly available due to hospital policy, but are available from the corresponding author on reasonable request.

## Abbreviations

MD: Medical Doctor
ABO: Acute Bowel Obstruction
MIS: Minimally Invasive Surgery
OS: Open Surgery
Gen Z: Generation Z
GI: Gastrointestinal
ASA: American Society of Anesthesiologists
WA: Washington State
USA: United States of America
BMI: Body Mass Index
CRP: C-reactive protein
LOS: Length of Stay
OR: Odds Ratio
CI: Confidence Interval
WSES: World Society of Emergency Surgery
RCT: Randomized Controlled Trial
EKS: Ethikkommission Salzburg
